# Seasonal forecasting of lightning and thunderstorm activity in tropical and temperate regions of the world

**DOI:** 10.1038/srep20874

**Published:** 2016-02-11

**Authors:** Andrew J. Dowdy

**Affiliations:** 1 Bureau of Meteorology, 700 Collins St, Docklands, VIC, 3008, Australia

## Abstract

Thunderstorms are convective systems characterised by the occurrence of lightning. Lightning and thunderstorm activity has been increasingly studied in recent years in relation to the El Niño/Southern Oscillation (ENSO) and various other large-scale modes of atmospheric and oceanic variability. Large-scale modes of variability can sometimes be predictable several months in advance, suggesting potential for seasonal forecasting of lightning and thunderstorm activity in various regions throughout the world. To investigate this possibility, seasonal lightning activity in the world’s tropical and temperate regions is examined here in relation to numerous different large-scale modes of variability. Of the seven modes of variability examined, ENSO has the strongest relationship with lightning activity during each individual season, with relatively little relationship for the other modes of variability. A measure of ENSO variability (the NINO3.4 index) is significantly correlated to local lightning activity at 53% of locations for one or more seasons throughout the year. Variations in atmospheric parameters commonly associated with thunderstorm activity are found to provide a plausible physical explanation for the variations in lightning activity associated with ENSO. It is demonstrated that there is potential for accurately predicting lightning and thunderstorm activity several months in advance in various regions throughout the world.

Thunderstorms can have a wide range of impacts on natural and built environments. The lightning associated with thunderstorms can directly result in loss of life and damage to infrastructure^1^,^2^,^3^,^4^, as well as lead to the occurrence of hazards such as wildfires^5^,^6^,^7^,^8^. Lightning can also influence atmospheric chemistry through its production of reactive nitrogen species^9^,^10^,^11^, important precursors for tropospheric hydroxyl and ozone^12^,^13^, as well as influence the occurrence of transient luminous events (such as sprites, elves, halos and electric jets)^14^. Thunderstorms can sometimes be associated with hazards such as extreme rainfall, winds, hail and tornado occurrence^15^,^16^. Thunderstorms are also a major driver of the global atmospheric distribution of water thereby influencing both long and short wave radiation^17^,^18^,^19^,^20^, with the global distribution of upper-tropospheric ice broadly consistent with thunderstorm behaviour^21^. Given the diverse range of impacts associated with thunderstorms, seasonal forecasts of thunderstorm activity could potentially have a wide range of benefits for groups such as industry, government, insurance and emergency services.

Despite the chaotic nature of weather^22^, the nonstationarity of large-scale modes of atmospheric and oceanic variability, such as ENSO, can lead to skill in future predictions of seasonal conditions in some cases^23^,^24^. For example, some severe weather phenomena associated with convective systems have been examined recently in relation to seasonal forecasting applications based on ENSO, including hail and tornado occurrence in the United States^15^,^16^. There are many examples of studies that have examined anomalous lightning and thunderstorm activity in relation to ENSO^25^,^26^,^27^,^28^,^29^,^30^,^31^,^32^,^33^,^34^,^35^,^36^,^37^ and other large-scale modes of variability such as the North Atlantic Oscillation (NAO)^38^, the Quasi-Biennial Oscillation (QBO)^39^ and the Indian Ocean Dipole^40^. However, seasonal forecasting of lightning and thunderstorm activity has not specifically been examined in the literature, noting that this is examined here throughout tropical and temperate regions of the world (in the study region from 35°N to 35°S).

Seasonal lightning activity is examined here in relation to various different large-scale modes of variability for an 18 year period from 1996 to 2013. Lightning flash data are obtained from two NASA satellite sensors: the Lightning Imaging Sensor (LIS) and the Optical Transient Detector (OTD)^41^,^42^. The lightning data are examined in relation to seven different modes of variability: the El Niño/Southern Oscillation^43^ (ENSO, as represented by the NINO3.4 index), an ocean-atmosphere coupled mode with strong interaction between the Walker Circulation and the Pacific Ocean; the Northern Annual Mode^44^ (NAM, also referred to as the Arctic Oscillation: AO) and the Southern Annular Mode^45^ (SAM), both of which are characterised by north-south shifts in atmospheric mass between the polar regions and the middle latitudes; the North Atlantic Oscillation^46^ (NAO), characterised by anomalous atmospheric pressure in the North Atlantic region; the Pacific-North American Pattern^46^ (PNA), characterised by anomalous strength and location of the East Asian jet stream; the Indian Ocean Dipole^47^ (IOD, as represented by the Dipole Mode Index, DMI), a coupled ocean-atmosphere phenomenon located in the tropical Indian Ocean; and the Quasi-Biennial Oscillation (QBO)^48^, a quasi-periodic variation in stratospheric zonal winds in the tropics.

Seasonal variations in a number of different atmospheric parameters commonly associated with thunderstorm activity are also examined, based on ERA-Interim reanalysis^49^. Results are examined in relation to the potential for seasonal forecasting of lightning and thunderstorm activity, including through the use of hindcast (i.e. reforecast) NINO3.4 values obtained from the National Oceanic and Atmospheric Administration (NOAA).

## Results

### Relationship to various modes of variability

Lightning flash densities (flashes km^−2^) are examined here for the study region from 35°N to 35°S within the field of view of both the satellite sensors. Table 1 shows the percentage of the study region that has a significant correlation (at the 95% confidence level) between lightning flash density and seven indices representing the different large-scale modes of variability: NINO3.4, NAM, SAM, NAO, PNA, DMI and QBO (as detailed in the Methods section). The correlations are calculated individually for each different mode of variability and for each grid point of the study region. Correlations are based on seasonal values throughout the study period (from 1996 to 2013) and are calculated individually for four seasons of the year: December, January and February (DJF), March, April and May (MAM), June, July and August (JJA) and September, October and November (SON).

In every one of the four seasons throughout the year, NINO3.4 is significantly related to lightning flash density at more locations than any of the other six indices (Table 1). Significant correlations for NINO3.4 occur at 15–24% of the total study region, depending on the season, with the largest value occurring during the SON season (24%). For DMI, significant correlations occur at 5–19% of the study region, with the largest value occurring during the SON season (19%). Other examples include significant correlations at 12% of the study region for PNA during the DJF season and at 11% of the study region for SAM during the SON season. Although there are some locations where NAM, NAO and QBO are significantly correlated with lightning flash density, ranging from 3–8% of the study region, it is noted that these values are relatively small given that 5% of the study region on average could be expected to have a significant correlation at the 95% confidence level based on random chance.

There are relatively few locations where lightning flash density is not significantly related to NINO3.4 but is significantly related to one of the other indices, with this occurring for 9% or less of the study region in all cases shown in Table 1. For the example of the DMI index during the SON season, 19% of the study region has a significant correlation, but only 5% of the study region has a significant correlation at locations that are different to the case for NINO3.4. This could be expected to some degree due to the strong relationship between DMI and NINO3.4 during the SON season (*r* = 0.68), as shown by the seasonal correlations between the seven different modes of variability listed in Supplementary Table 1. Many of the other indices show a considerably higher degree of correlation with each other: NAM and NAO is the pair of indices that are most closely related to each other, with *r* ≥ 0.65 in every individual season, noting that previous studies have also shown strong similarities between these two modes of variability^44^.

### Spatial and seasonal variability of the relationships

ENSO has the strongest and most widespread relationship to lightning activity of the seven modes of variability examined here, hence hereafter the focus of this study is primarily on this relationship. Large positive anomalies of the NINO3.4 index are typically associated with El Niño, the warm phase of ENSO, characterised by above average sea-surface temperatures in the eastern Pacific and higher air pressures in the western Pacific than the eastern Pacific^43^,^50^. Large magnitude negative anomalies of the NINO3.4 index are typically associated with La Niña, the cold phase of ENSO, characterised by below average sea-surface temperatures in the eastern Pacific and higher air pressures in the eastern Pacific than the western Pacific.

**Figure 1:**
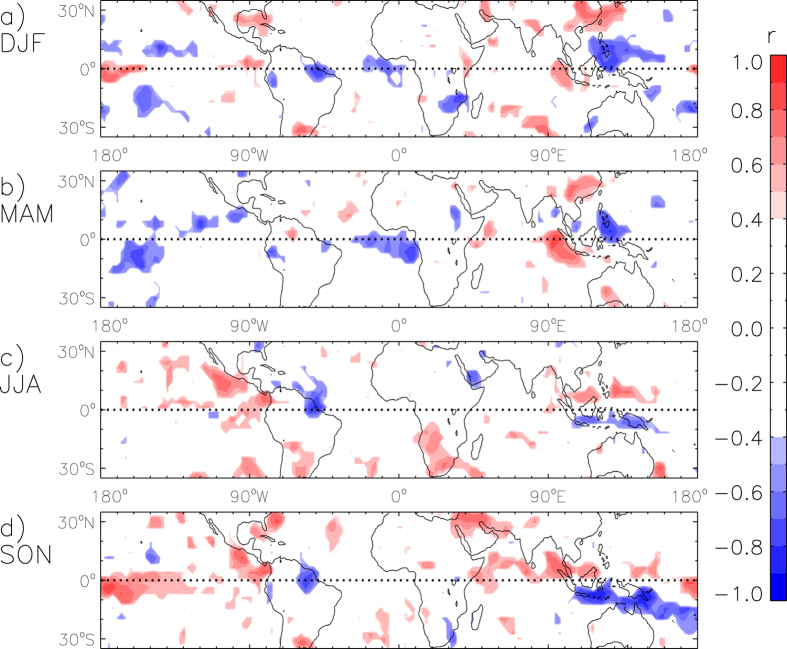
Correlations between seasonal lightning flash density and NINO3.4, for the time period from 1996 to 2013. The correlations are shown for locations where the relationship is significant at the 95% confidence level, calculated individually for each season: DJF (**a**), MAM (**b**), JJA (**c**) and SON (**d**). Coastlines are shown based on the land-sea mask of the ERA-Interim reanalysis, with data visualisations produced using IDL 8.3 (Exelis Visual Information Solutions, Boulder, Colorado).

Spatial maps of the correlation between NINO3.4 and lightning flash densities throughout the study region are shown individually for each of the four seasons in Fig. 1. The correlations are shown for locations where the relationship is significant at the 95% confidence level.

Considerable variations occur throughout the year in the relationship between ENSO and seasonal lightning activity. The NINO3.4 index is significantly related to lightning activity at 53% of all grid-point locations for one or more seasons throughout the year, noting that about 19% of the study region on average could be expected to have a significant correlation for at least one or more seasons at the 95% confidence level based on random chance. 50% of land locations and 54% of ocean locations have a significant correlation between lightning activity and NINO3.4 for one or more seasons, noting that about two thirds of the study region is oceanic. For land regions, a significant positive correlation occurs for one or more seasons at 35% of locations, and a significant negative correlation occurs at 18% of locations. Similarly for ocean regions, a significant positive correlation occurs for one or more seasons at 37% of locations, and a significant negative correlation occurs at 23% of locations.

Maps similar to Fig. 1, but for the 6 indices other than NINO3.4 (i.e. NAM, SAM, NAO, PNA, DMI and QBO), are shown in Supplementary Figures 1–6. These maps show relatively few locations where significant correlations occur at locations different to the case for NINO3.4. Although there are some regional exceptions to this, such as for the DMI in SON in the equatorial Indian Ocean (Supplementary Figure 5), the percentage of the study region for which this occurs is similar to what would be expected based on random chance alone (as noted previously from Table 1).

Modes of variability such as NAM and SAM are characterised by shifts in atmospheric mass between polar and mid-latitude regions, such that associated teleconnections may take some time to become apparent in locations away from their region of origin, noting that the majority of lightning activity occurs in the tropics^51^,^52^. However, lagged correlations based on lightning activity one season in advance (i.e. 3 months later) with respect to NAM or SAM show few regions where significant relationships exist (Supplementary Figures 7 and 8), as is also the case for NAO, PNA, DMI and QBO (Supplementary Figures 9–12). In contrast, lagged correlations based on lightning activity one season in advance of the NINO3.4 index (Supplementary Figure 13) are broadly similar to the unlagged correlations (Fig. 1) with the exception of JJA for which there are relatively few locations where significant correlations exist (consistent with the lack of persistence in ENSO conditions at this time of year^53^,^54^).

### An examination of four cases

**Figure 2:**
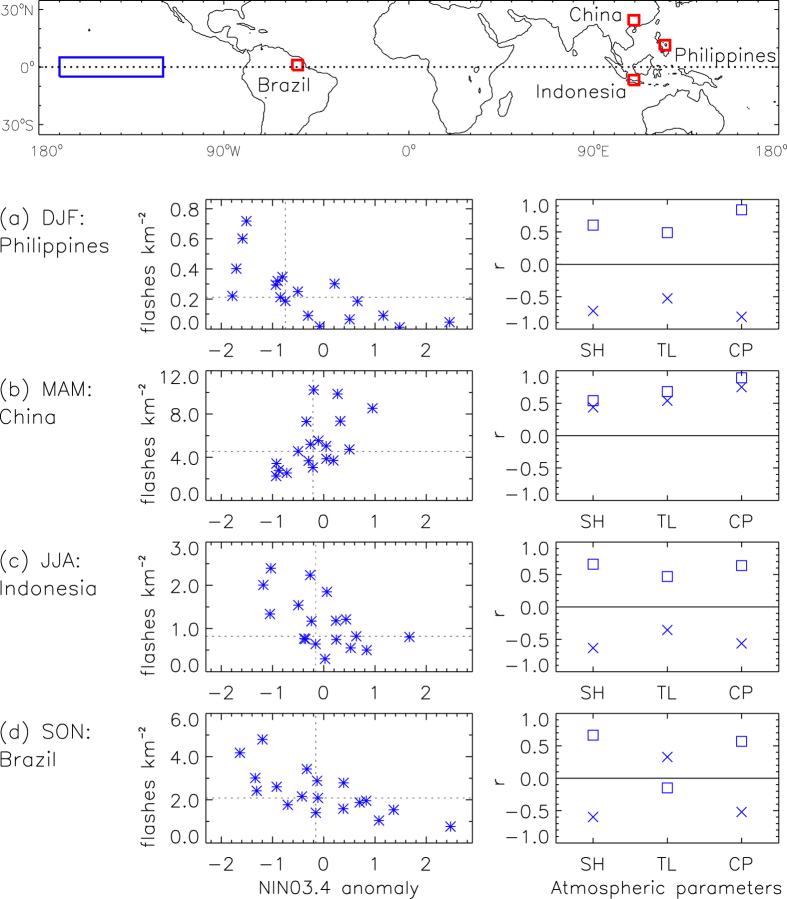
The influence of ENSO on lightning and thunderstorm characteristics for four different regions and seasons, for the time period from 1996 to 2013. The four regions are bounded by the red boxes shown in the map (upper panel) and are located in the Philippines for DJF (**a**), China for MAM (**b**), Indonesia for JJA (**c**) and Brazil for SON (**d**). Daily lightning flash density versus NINO3.4 is shown (left panels), with median values of lightning flash density (horizontal dotted lines) and median values of NINO3.4 (vertical dotted lines). Correlation coefficients are shown (right panels, ‘x’ symbols) for the relationships between NINO3.4 and three atmospheric parameters (based on reanalysis) associated with thunderstorm occurrence: specific humidity (SH), temperature lapse (TL) and convective available potential energy (CP). Correlation coefficients are also shown (right panels, ‘□’ symbols) for the relationships between lightning flash density and the three atmospheric parameters. The NINO3.4 region is shown in blue (upper panel: from 5°N to 5°S in latitude and from 120°W to 170°W in longitude).

Figure 2 investigates the influence of ENSO for four individual regions and seasons for the time period from 1996 to 2013. These four examples were selected due to having strong relationships between lightning activity and ENSO, and are located in the Philippines for the DJF season, China for the MAM season, Indonesia for the JJA season and Brazil for the SON season. The regions are based on individual grid points of the lightning data, noting that each grid point represents the mean value for a region spanning 7.5 degrees in both latitude and longitude (as shown by the red squares in Fig. 2, upper panel).

Lightning flash densities versus NINO3.4 anomalies are shown in Fig. 2 (left panels). Also presented in Fig. 2 (right panels) is an examination of NINO3.4 and lightning flash density in relation to three different atmospheric parameters associated with favourable environments for thunderstorm occurrence: specific humidity at 925 hPa (SH), temperature lapse at 850-500 hPa (TL) and surface-based Convective Available Potential Energy (CAPE: CP). The parameters are based on ERA-Interim reanalysis^49^ and were selected to represent commonly used measures of moisture, heat and momentum in the atmosphere. These three parameters are not expected to entirely explain the exact physical cause of variations in lightning activity throughout the world as there are a large number of different factors that can potentially influence convective storm development^16^,^27^,^28^,^36^,^52^,^55^,^56^,^57^,^58^,^59^.

The atmospheric parameters are strongly correlated with NINO3.4 in many cases, as well as with lightning flash density. This is the case for specific humidity and CAPE in all four locations shown in Fig. 2 as well as for temperature lapse in the Philippines and China examples. The sign of these correlations is consistent with what would be expected if ENSO-related variations in these parameters were influencing lightning activity, thereby providing a degree of physical plausibility for the relationships between ENSO and lightning activity. For example, the results for the Philippines in DJF (Fig. 2a) show that lightning activity and specific humidity are both typically lower than average during El Niño conditions (i.e., negatively correlated with NINO3.4), with a positive correlation between specific humidity and lightning activity. Temperature lapse has a somewhat stronger relationship to ENSO and lightning flash density for the Philippines and China cases than for the Indonesia and Brazil cases, with this being similar to the case for CAPE (which could be expected to some degree given that CAPE can be influenced by temperature lapse rate as well as by atmospheric moisture content).

### Comparison with previous studies

A number of previous studies have examined the ENSO/lightning relationship, particularly for the 1997–1998 El Niño event and its influence on lightning activity in the Maritime Continent region. For example, one study^26^ noted more lightning activity over Indonesia during March 1998 (El Niño conditions) than March 1999 (La Niña conditions). The region examined in that study encompasses the region examined here in Fig. 2c (centered over the island of Java, Indonesia). This is an example of a location where significant correlations with NINO3.4 occur of opposite sign for different times of the year: the sign of the correlation is positive during the first half of the year (DJF and MAM) and negative during the second half of the year (JJA and SON) as shown here in Fig. 1. To examine this example in more detail and allow greater comparison to be made with previous studies, Supplementary Figure 14 details the seasonal variation in the lightning/ENSO relationship for this region. The lightning flash densities during the 1998 El Niño event are the highest on record for MAM (as well as for the preceding season, DJF) based on the entire 18-year period of available observations for this region. The results shown here for this region are therefore similar to the conclusions presented by that previous study^26^, of increased lightning activity in the 1998 El Niño case, while noting that the previous study focussed on data for only two months (March 1998 and March 1999) in contrast to the 18 years of MAM seasonal values as used here.

Another previous study^28^ also reported increased lightning activity in the Indonesian region for El Niño conditions, based on the average for two different time periods (for the time period from January to August 1998 combined with the time period from March 2002 to February 2003). This combined time period weights the first part of the year twice as highly as the last part of the year. Consequently, the average values for this combined time period could be expected to indicate increased lightning activity for El Niño conditions, due in part to the positive correlations with NINO3.4 that occur in this region during the start of the year (see Fig. 1, as well as Supplementary Figure 14) while also noting the larger magnitude of the lightning flash density during the start of the year compared with the end of the year in this region (Supplementary Figure 14).

The study region examined by that previous study^28^ as well as by another similar study^34^ encompasses the location in eastern China that was examined here in Fig. 2b. The results presented in these two previous studies indicate increased lightning activity for El Niño events in this eastern China region. With broad similarity to these previous studies, Supplementary Figure 15 shows that more lightning activity tends to occur during El Niño than La Niña conditions in this region due to the relatively strong ENSO influence on lightning activity during the first half of the year (DJF and MAM), with relatively little ENSO influence on lightning activity during the second half of the year (JJA and SON). It also shows that the lightning activity in this region during the 1998 El Niño was the highest on record for DFJ throughout the 18-year study period and third highest on record for MAM.

One previous study^33^ examined global lightning flash counts in relation to ENSO with a focus on two 12-month time periods to define El Niño conditions (during 1997/1998 and 2002/2003) and two 12-month time periods to define La Niña conditions (during 1995/1996 and 1998/1999), indicating about 10% more lightning globally for El Niño than La Niña conditions. That study also examined individual regions in relation to lightning variability associated with ENSO, such as in their Fig. 8 for South America which indicated more lightning for El Niño than for La Niña conditions at around 30°S, while at around 10°S more lightning occurs for La Niña than El Niño conditions. This change in sign of the ENSO/lightning relationship in South America is broadly consistent with the results presented here in Fig. 1, while noting various differences in method between their study and this study (such as their use of average lightning activity over large regions, as well as their use of 12-month time periods to examine specific El Niño and La Niña events). It is also noted that there can be considerable differences in the lightning response between individual ENSO events, as is apparent between two different years they classified as La Niña in their Figure 9 for the Ganges River valley.

In addition to these previous studies which use a case study type of approach to examine the ENSO/lightning relationship (i.e., examining a number of individual ENSO events and/or individual regions), a recent study^29^ applied a more systematic approach to examine this relationship. That study examined the ENSO/lightning relationship for each individual location globally (based on the OTD/LIS sensor data coverage) and for all times throughout their study period (from 1997 to 2006). Their results show that there is considerable spatial variation in the magnitude and sign of the relationship, based on examining the ENSO/lightning relationship for all time periods combined throughout the entire year (rather than for individual seasons). Building on the results of previous studies such as these mentioned above, the results presented here examine for the first time the seasonal variation in the ENSO/lightning relationship (in addition to the spatial variability in this relationship), thereby allowing an investigation of the potential for seasonal forecasting of lightning and thunderstorm activity throughout tropical and temperate regions of the world.

### Potential for seasonal forecasting

Hindcasts (i.e., reforecasts) of NINO3.4 values were obtained from NOAA for the period 1996 to 2010, with a three-month lead time prior to the start of each season^60^. These hindcasts are used here to examine predictions of lightning flash density as either higher or lower than their seasonal median value, based on whether the hindcast NINO3.4 values are higher or lower than their seasonal median value. The accuracy of the predictions is calculated as the number of correct predictions divided by the total number of predictions.

**Figure 3:**
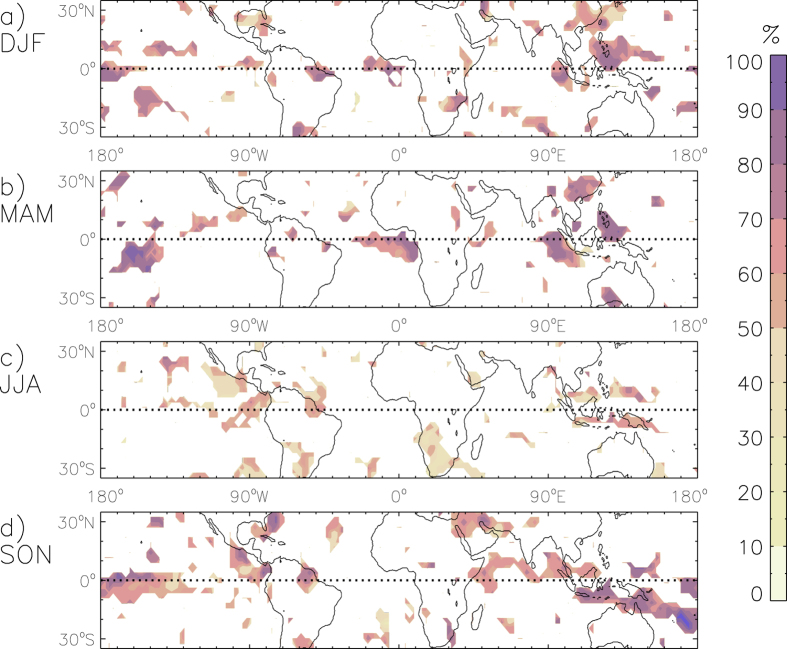
Seasonal prediction of lightning flash density with a lead time of 3 months. The accuracy of the forecast is shown here as the percentage number of predictions that are correct, based on predictions of lightning flash density as either above or below the seasonal median value, for the time period from 1996 to 2010. This is shown for locations where a significant relationship exists between lightning activity and the NINO3.4 index (from Fig. 1). This is presented for each of the four seasons: DJF (**a**), MAM (**b**), JJA (**c**) and SON (**d**).

Figure 3 shows the accuracy of the predictions, presented for all locations where a significant relationship exists between lightning activity and ENSO (based on Fig. 1). The accuracy is higher for DJF, MAM and SON than for JJA: the mean value of the accuracy is 52% for JJA as shown in Fig. 3 (i.e. similar to that of random chance) compared with a mean value of 64–70% for each of the other three seasons. There are some regions and seasons where the accuracy shown in Fig. 3 is higher than 70%, including for three of the four examples examined previously from Fig. 2 (Philippines for DJF, China for MAM and Brazil for SON), with relatively low accuracy of 53% for one of those four examples (JJA in Indonesia). The low accuracy during JJA is associated with a period of low hindcast skill for NINO3.4 in the months preceding the JJA season (i.e. March, April and May)^60^ and is an example of the challenges that remain in understanding ENSO variability and predictability.

### Anomaly maps for ENSO phases

**Figure 4:**
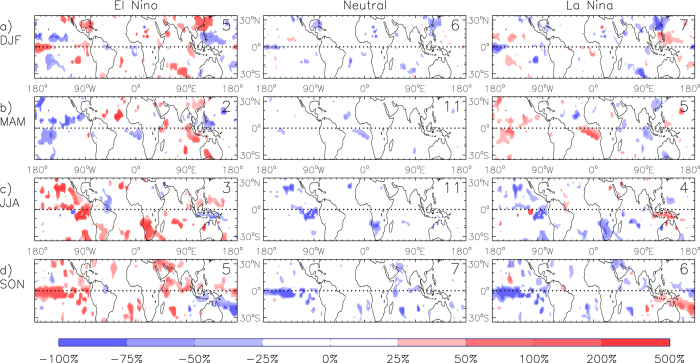
Anomalous lightning activity for various ENSO phases, for the time period from 1996 to 2013. Average seasonal anomalies (percentage difference to the seasonal mean) of lightning flash density are shown for various phases of ENSO: El Niño (left panels), Neutral (central panels) and La Niña (right panels) events. The number of events used to create the average for each case is shown in the top right corner of each panel. Anomalies are shown at locations where significant correlations occur between lightning flash density and NINO3.4 (at the 95% confidence level). This is presented for each of the four seasons: DJF (**a**), MAM (**b**), JJA (**c**) and SON (**d**).

Figure 4 presents seasonal anomalies of lightning flash densities for three different phases of ENSO (El Niño, Neutral and La Niña), shown for locations where a significant relationship occurs (from Fig. 1). The anomalies are calculated as the percentage difference to the seasonal mean at each individual grid-point. A benefit of examining these anomaly fields (Fig. 4) is that they show the relative magnitude of the variation in lightning activity associated with ENSO, in contrast to the correlations (Fig. 1) which indicate the strength of the relationship between lightning activity and ENSO. The phases of ENSO are defined using three-month average values of NINO3.4 with values greater (less) than or equal to 0.5 (−0.5) defined as El Niño (La Niña) conditions if this occurs for at least two consecutive seasons, otherwise the conditions are defined as Neutral (similar to the convention used by NOAA^61^). The period of data available for this study is such that each of these three different ENSO phases occurs at least twice during MAM and JJA and at least five times during DJF and SON (as listed in Fig. 4 and Supplementary Table 2).

The lightning anomalies tend to be larger in magnitude for the two extreme phases of ENSO (i.e. El Niño and La Niña) than for the Neutral phase. Large regions with positive anomalies are particularly apparent in the cases shown for El Niño conditions, while the anomalies for neutral conditions are predominantly negative. The lightning anomalies are of the order of 25% to 75% of the seasonal average in many cases for the El Niño and La Niña phases.

Figure 4 could be used to provide guidance in relation to seasonal forecasting applications. For example, if El Niño conditions were forecast to occur for an upcoming DJF season, then it might be expected that the southeast China region could experience increased lightning activity, while the region near the Philippines could experience decreased lightning activity. Similarly, if El Niño conditions were forecast to occur for an upcoming SON season, then it might be expected that the equatorial Central Pacific region could experience increased lightning activity, while the western South Pacific region could experience decreased lightning activity.

## Discussion

Of the seven different modes of variability examined here, ENSO was found to have the strongest relationship with lightning activity during each individual season, with relatively little relationship for the other modes of variability. ENSO is the strongest source of atmospheric and oceanic variability throughout the world at interannual time scales and provides a large source of seasonal forecasting skill^16^,^23^,^24^,^62^. Although ENSO originates in the tropical Pacific Ocean region, it can affect land and ocean regions in many regions throughout the world, including through the modulation of phenomena such as the Walker Circulation, extratropical storm tracks, jetstreams and the production of Rossby wave trains that propagate to mid- and high-latitudes^17^,^63^,^64^,^65^,^66^,^67^.

There is considerable evidence that variations in lightning and thunderstorm activity can be related to ENSO in some cases. For example, a number of previous studies examined lightning activity in relation to particular ENSO events, including many reports of anomalous lightning activity for the 1997–98 El Niño and subsequent La Niña conditions^20^,^25^,^26^,^27^,^31^,^32^,^33^,^34^. In particular, the Maritime Continent region of Southeast Asia has been identified previously, as well as here, as being a region where strong relationships exist between lightning activity and ENSO^26^,^28^,^29^,^32^,^33^,^34^,^68^. Other examples of strong relationships, as noted here and in previous studies, include northeast Brazil^29^,^37^, the western North Pacific region^25^,^27^,^28^,^29^ and the Gulf of Mexico^25^,^33^.

Variations in atmospheric parameters associated with thunderstorm activity were found to provide a plausible physical explanation for the ENSO-related lightning variability. A method for predicting anomalous lightning and thunderstorm activity was examined, while noting that there is a wide range of methods that could potentially be used for seasonal forecasting applications, including both statistical and dynamical modelling techniques. The accuracy of the seasonal predictions was found to be better for DJF, MAM and SON than for JJA. The relative weakness of the skill for JJA could be expected to some degree given that this is the typical transition period for ENSO signals.

An ability to predict variations in lightning and thunderstorm activity could be expected to lead to improved preparedness for associated impacts on built and natural environments. Given the wide range of impacts associated with lightning and thunderstorm activity throughout the world, this could have benefits for groups such as industry, government, insurance and emergency services. The results presented here show that there is potential for accounting for, and also predicting in advance, the variability of seasonal lightning and thunderstorm activity in various regions of the world.

## Methods

### Lightning data

The lightning flash data used here are based on observations from two NASA satellite sensors: the Lightning Imaging Sensor (LIS) and the Optical Transient Detector (OTD)^41^,^42^. The Optical Transient Detector (OTD) was launched into low Earth orbit in April 1995 on the OV-1 satellite (formerly MicroLab-1), with this mission ending in March 2000, such that the total period of available data covers about 5 years. The Lightning Imaging Sensor (LIS) on the Tropical Rainfall Measuring Mission (TRMM) satellite^69^ was launched in December 1997 and ceased operation in April 2015. Due to its orbit, the LIS sensor provided coverage only within the region from about 38°S to 38°N. Details of the seasonal climatology of the LIS and OTD data were recently presented^70^. The 18-year period of data available for this study spans multiple occurrences of different phases of the various large-scale modes of variability examined here.

Lightning flash densities throughout the time period from 1996 to 2013 were obtained from the monthly gridded time series product (name “LISOTD_LRMTS”, version “V2.3.2014”) produced from a combination of the LIS and OTD data (available from http://thunder.nsstc.nasa.gov)^71^. The spatial resolution of the data is 2.5 degrees in both latitude and longitude, although prior to being released the data are smoothed with a 7.5° × 7.5° and approximately 3 month (111 days for OTD and 99 days for LIS) boxcar moving average (i.e. running mean) due to the approximate 3 month revisit time of the satellite coverage through the diurnal cycle at individual locations. Due to the effective temporal resolution of the satellite data (based on the approximate 3 month moving average), seasonal lightning activity is examined here based on the data for January (to represent the December, January and February season: DJF), April (to represent the March, April and May season: MAM), July (to represent the June, July and August season: JJA) and October (to represent the September, October and November season: SON). In contrast to this method, if three consecutive months of the data were averaged to try to represent seasonal lightning activity, it would actually represent lightning activity over a time period much longer than one season (i.e., a time period of over 5 months). The geographic study region used here covers all longitudes globally, and ranges in latitude from 35°S to 35°N within the field of view of both the OTD and LIS sensors, based on a 2.5 degree grid in both latitude and longitude.

### Modes of variability

Seven different indices are used in this study, representing seven different large-scale modes of variability. Six of these indices were obtained from the National Oceanic and Atmospheric Administration (NOAA) (from http://www.cpc.ncep.noaa.gov/, accessed January 2015), including representations of the El Niño/Southern Oscillation^43^ (based on NINO3.4 anomalies), the Northern Annular Mode^44^ (NAM), the North Atlantic Oscillation^46^ (NAO), the Pacific-North American Pattern^46^ (PNA), the Indian Ocean Dipole^47^ (based on the Dipole Mode Index, DMI) and the Quasi-Biennial Oscillation^48^ (QBO, based on the 50 hPa zonal wind). A seventh index representing the Southern Annular Mode^45^ (SAM) is used as provided by the British Antarctic Survey^72^. These seven large-scale modes of variability have a period of variability less than decadal and greater than monthly, as required given that the period of available lightning data is not long enough to allow decadal variations to be examined and that the effective temporal resolution of the lightning data is about 3 months. The seven indices used here represent commonly used measures of these modes of variability. Three month averages of these indices are used here for DJF, MAM, JJA and SON.

### Reanalysis

ERA-Interim reanalysis^49^ is used to derive three different atmospheric parameters, including specific humidity at 925 hPa, temperature lapse at 850-500 hPa and surface-based Convective Available Potential Energy (CAPE). Seasonal averages are produced for DJF, MAM, JJA and SON, with a grid spacing of 7.5 degrees in both latitude and longitude, consistent with that of the lightning data. Coastlines are shown in figures presented here based on the land-sea mask of the ERA-Interim reanalysis.

### ENSO forecasts

Hindcasts (i.e., reforecasts) of monthly NINO3.4 values were obtained from NOAA for the period December 1995 to November 2010 (from http://nomads.ncdc.noaa.gov/modeldata/ accessed January 2015), based on forecasts of the tropical Pacific sea surface temperatures using a linear statistical model (Markov model) trained for the 1981–1998 period^60^. Seasonal average values are used here for DJF, MAM, JJA and SON, with the hindcast values based on a three-month lead time prior to the start of a season (e.g., values for the DJF season are based on initial conditions for the preceding September).

### Statistical analyses

The sample Pearson’s correlation coefficient, *r*, is used to examine the dependence between datasets. The 95% confidence level is used to examine the significance of results, determined using a nonparametric bootstrap method based on 10 000 random permutations of the data, with two-sided confidence intervals based on percentiles.

## Additional Information

**How to cite this article**: Dowdy, A. J. Seasonal forecasting of lightning and thunderstorm activity in tropical and temperate regions of the world. *Sci. Rep.* **6**, 20874; doi: 10.1038/srep20874 (2016).

## Supplementary Material

Supplementary Information

## Figures and Tables

**Table 1:** The percentage of the study region that has a significant correlation at the 95% confidence level between seasonal lightning flash density and seven different large-scale modes of variability.

Index	DJF	MAM	JJA	SON
NINO3.4	21	15	18	24
NAM	6 (3)	4 (4)	5 (4)	5 (4)
SAM	4 (2)	3 (3)	4 (3)	11 (6)
NAO	7 (4)	6 (5)	6 (5)	4 (3)
PNA	12 (6)	6 (6)	6 (5)	4 (3)
DMI	15 (9)	5 (4)	10 (7)	19 (5)
QBO	4 (3)	3 (2)	7 (5)	8 (5)

The indices representing the large-scale modes of variability are NINO3.4, NAM, SAM, NAO, PNA, DMI and QBO. Results are shown for four individual seasons (DJF, MAM, JJA and SON), based on the time period from 1996 to 2013. The study region is from 35°N to 35°S, globally. The values in the brackets represent the percentage of the study region for which the correlation is significant for a given index but is not significant for the NINO3.4 index.
