# Supplementary material for: Seasonal forecasting of lightning and thunderstorm activity in tropical and temperate regions of the world
Source: Sci Rep. 2016 Feb 11;6:20874. doi: 10.1038/srep20874 (PMC4750006; doi:10.1038/srep20874)
Supplement: Supplementary Information [file srep20874-s1.doc]

**Supplementary information:**

**Seasonal forecasting of lightning and thunderstorm activity in tropical and temperate regions of the world**

Andrew J. Dowdy

**Supplementary Table 1 | The correlation between different large-scale modes of variability.** This is shown for different pairs of indices representing the modes of variability (for NINO3.4, DMI, SAM, NAM, NAO, PNA and QBO) and for each of the four seasons (DJF, MAM, JJA and SON), based on seasonal averages for each individual year over the study period (from 1996 to 2013).

| Index | DJF | MAM | JJA | SON |
| --- | --- | --- | --- | --- |
| NINO3.4 and DMI | 0.49 | -0.31 | 0.32 | 0.68 |
| NINO3.4 and SAM | -0.36 | -0.01 | -0.06 | -0.49 |
| NINO3.4 and NAM | -0.44 | -0.01 | -0.23 | -0.16 |
| NINO3.4 and NAO | -0.41 | -0.26 | 0.12 | -0.30 |
| NINO3.4 and PNA | 0.63 | 0.05 | -0.13 | -0.24 |
| NINO3.4 and QBO | 0.29 | -0.09 | 0.39 | 0.32 |
| DMI and SAM | -0.06 | 0.01 | 0.15 | -0.42 |
| DMI and NAM | -0.31 | 0.53 | -0.48 | 0.05 |
| DMI and NAO | -0.39 | 0.35 | -0.49 | -0.08 |
| DMI and PNA | 0.35 | 0.19 | -0.03 | -0.22 |
| DMI and QBO | 0.40 | 0.33 | 0.28 | 0.46 |
| SAM and NAM | 0.76 | -0.41 | 0.01 | 0.16 |
| SAM and NAO | 0.57 | -0.22 | -0.42 | -0.01 |
| SAM and PNA | -0.42 | 0.33 | 0.06 | 0.24 |
| SAM and QBO | 0.13 | -0.17 | -0.23 | -0.16 |
| NAM and NAO | 0.85 | 0.76 | 0.65 | 0.74 |
| NAM and PNA | -0.25 | -0.41 | -0.41 | -0.16 |
| NAM and QBO | 0.06 | 0.58 | -0.35 | 0.20 |
| NAO and PNA | -0.03 | -0.49 | -0.18 | 0.25 |
| NAO and QBO | -0.05 | 0.62 | 0.12 | 0.10 |
| PNA and QBO | 0.00 | -0.01 | -0.10 | -0.06 |

**Supplementary Table 2 | ENSO phases throughout the study period. Seasonal average values of NINO3.4 are shown, with red representing El Niño, black representing Neutral and blue representing La Niña conditions. This is shown for the four seasons of DJF, MAM, JJA and SON (the year shown in the left column for the DJF season is based on the year for January and February rather than the year for December).**

| Year | DJF | MAM | JJA | SON |
| --- | --- | --- | --- | --- |
| 1996 | **-0.9** | **-0.5** | **-0.2** | **-0.4** |
| 1997 | **-0.5** | **0.3** | **1.7** | **2.5** |
| 1998 | **2.5** | **1.0** | **-1.2** | **-1.3** |
| 1999 | **-1.6** | **-0.9** | **-1.1** | **-1.2** |
| 2000 | **-1.7** | **-0.9** | **-0.5** | **-0.7** |
| 2001 | **-0.8** | **-0.3** | **0.1** | **-0.2** |
| 2002 | **-0.1** | **0.2** | **0.8** | **1.4** |
| 2003 | **1.2** | **0.0** | **0.0** | **0.4** |
| 2004 | **0.2** | **0.0** | **0.4** | **0.7** |
| 2005 | **0.5** | **0.3** | **0.2** | **-0.1** |
| 2006 | **-0.8** | **-0.3** | **0.2** | **0.8** |
| 2007 | **0.7** | **-0.1** | **-0.4** | **-1.3** |
| 2008 | **-1.8** | **-0.9** | **-0.2** | **-0.3** |
| 2009 | **-0.8** | **-0.2** | **0.6** | **1.1** |
| 2010 | **1.5** | **0.5** | **-1.0** | **-1.6** |
| 2011 | **-1.5** | **-0.7** | **-0.4** | **-0.9** |
| 2012 | **-0.9** | **-0.3** | **0.5** | **0.4** |
| 2013 | **-0.3** | **-0.2** | **-0.3** | **-0.1** |

The following figures are based on the seven different indices used in this study to represent the seven different large-scale modes of variability (as detailed in the Methods section of the primary manuscript) including NINO3.4 (for the El Niño/Southern Oscillation: ENSO), NAM (Northern Annular Mode), NAO (North Atlantic Oscillation), PNA (Pacific-North American Pattern), DMI (Dipole Mode Index, for the Indian Ocean Dipole: IOD), QBO (Quasi-Biennial Oscillation) and SAM (Southern Annular Mode).

**Supplementary Figure 1 | Correlations between seasonal lightning flash density and NAM, for the time period from 1996 to 2013**. The correlations are calculated individually for each season: DJF (a), MAM (b), JJA (c) and SON (d). The coloured regions represent locations where the correlations are significant at the 95% confidence level. Coastlines are shown based on the land-sea mask of the ERA-Interim reanalysis, with data visualisations produced using IDL 8.3 (Exelis Visual Information Solutions, Boulder, Colorado).

**Supplementary Figure 2 | Correlations between seasonal lightning flash density and SAM, for the time period from 1996 to 2013**. The correlations are calculated individually for each season: DJF (a), MAM (b), JJA (c) and SON (d). The coloured regions represent locations where the correlations are significant at the 95% confidence level. Coastlines are shown based on the land-sea mask of the ERA-Interim reanalysis, with data visualisations produced using IDL 8.3 (Exelis Visual Information Solutions, Boulder, Colorado).

**Supplementary Figure 3 | Correlations between seasonal lightning flash density and NAO, for the time period from 1996 to 2013**. The correlations are calculated individually for each season: DJF (a), MAM (b), JJA (c) and SON (d). The coloured regions represent locations where the correlations are significant at the 95% confidence level. Coastlines are shown based on the land-sea mask of the ERA-Interim reanalysis, with data visualisations produced using IDL 8.3 (Exelis Visual Information Solutions, Boulder, Colorado).

**Supplementary Figure 4 | Correlations between seasonal lightning flash density and PNA, for the time period from 1996 to 2013**. The correlations are calculated individually for each season: DJF (a), MAM (b), JJA (c) and SON (d). The coloured regions represent locations where the correlations are significant at the 95% confidence level. Coastlines are shown based on the land-sea mask of the ERA-Interim reanalysis, with data visualisations produced using IDL 8.3 (Exelis Visual Information Solutions, Boulder, Colorado).

**Supplementary Figure 5 | Correlations between seasonal lightning flash density and DMI, for the time period from 1996 to 2013**. The correlations are calculated individually for each season: DJF (a), MAM (b), JJA (c) and SON (d). The coloured regions represent locations where the correlations are significant at the 95% confidence level. Coastlines are shown based on the land-sea mask of the ERA-Interim reanalysis, with data visualisations produced using IDL 8.3 (Exelis Visual Information Solutions, Boulder, Colorado).

**Supplementary Figure 6 | Correlations between seasonal lightning flash density and QBO, for the time period from 1996 to 2013**. The correlations are calculated individually for each season: DJF (a), MAM (b), JJA (c) and SON (d). The coloured regions represent locations where the correlations are significant at the 95% confidence level. Coastlines are shown based on the land-sea mask of the ERA-Interim reanalysis, with data visualisations produced using IDL 8.3 (Exelis Visual Information Solutions, Boulder, Colorado).

**Supplementary Figure 7 | Correlations between seasonal lightning flash density and NAM values for the previous season, for the time period from 1996 to 2013**. The correlations are calculated individually for each season: DJF (a), MAM (b), JJA (c) and SON (d). The coloured regions represent locations where the correlations are significant at the 95% confidence level. Coastlines are shown based on the land-sea mask of the ERA-Interim reanalysis, with data visualisations produced using IDL 8.3 (Exelis Visual Information Solutions, Boulder, Colorado).

**Supplementary Figure 8 | Correlations between seasonal lightning flash density and SAM values for the previous season, for the time period from 1996 to 2013**. The correlations are calculated individually for each season: DJF (a), MAM (b), JJA (c) and SON (d). The coloured regions represent locations where the correlations are significant at the 95% confidence level. Coastlines are shown based on the land-sea mask of the ERA-Interim reanalysis, with data visualisations produced using IDL 8.3 (Exelis Visual Information Solutions, Boulder, Colorado).

**Supplementary Figure 9 | Correlations between seasonal lightning flash density and NAO values for the previous season, for the time period from 1996 to 2013**. The correlations are calculated individually for each season: DJF (a), MAM (b), JJA (c) and SON (d). The coloured regions represent locations where the correlations are significant at the 95% confidence level. Coastlines are shown based on the land-sea mask of the ERA-Interim reanalysis, with data visualisations produced using IDL 8.3 (Exelis Visual Information Solutions, Boulder, Colorado).

**Supplementary Figure 10 | Correlations between seasonal lightning flash density and PNA values for the previous season, for the time period from 1996 to 2013**. The correlations are calculated individually for each season: DJF (a), MAM (b), JJA (c) and SON (d). The coloured regions represent locations where the correlations are significant at the 95% confidence level. Coastlines are shown based on the land-sea mask of the ERA-Interim reanalysis, with data visualisations produced using IDL 8.3 (Exelis Visual Information Solutions, Boulder, Colorado).

**Supplementary Figure 11 | Correlations between seasonal lightning flash density and DMI values for the previous season, for the time period from 1996 to 2013**. The correlations are calculated individually for each season: DJF (a), MAM (b), JJA (c) and SON (d). The coloured regions represent locations where the correlations are significant at the 95% confidence level. Coastlines are shown based on the land-sea mask of the ERA-Interim reanalysis, with data visualisations produced using IDL 8.3 (Exelis Visual Information Solutions, Boulder, Colorado).

**Supplementary Figure 12 | Correlations between seasonal lightning flash density and QBO values for the previous season, for the time period from 1996 to 2013**. The correlations are calculated individually for each season: DJF (a), MAM (b), JJA (c) and SON (d). The coloured regions represent locations where the correlations are significant at the 95% confidence level. Coastlines are shown based on the land-sea mask of the ERA-Interim reanalysis, with data visualisations produced using IDL 8.3 (Exelis Visual Information Solutions, Boulder, Colorado).

**Supplementary Figure 13 | Correlations between seasonal lightning flash density and NINO3.4 values for the previous season, for the time period from 1996 to 2013**. The correlations are calculated individually for each season: DJF (a), MAM (b), JJA (c) and SON (d). The coloured regions represent locations where the correlations are significant at the 95% confidence level. Coastlines are shown based on the land-sea mask of the ERA-Interim reanalysis, with data visualisations produced using IDL 8.3 (Exelis Visual Information Solutions, Boulder, Colorado).

**Supplementary Figure 14 | The influence of ENSO on lightning and thunderstorm characteristics over Java, Indonesia, for the time period from 1996 to 2013.** The region is bounded by the red box shown in the map (upper panel) and examined here for DJF (a), MAM (b), JJA (c) and SON (d). Daily lightning flash density versus NINO3.4 is shown (left panels: red asterisks for 1998), with median values of lightning flash density (horizontal dotted lines) and median values of NINO3.4 (vertical dotted lines). Correlation coefficients are shown (right panels, 'x' symbols) for the relationships between NINO3.4 and three atmospheric parameters (based on reanalysis) associated with thunderstorm occurrence: specific humidity (SH), temperature lapse (TL) and convective available potential energy (CP). Correlation coefficients are also shown (right panels, '□' symbols) for the relationships between lightning flash density and the three atmospheric parameters. Coastlines are shown based on the land-sea mask of the ERA-Interim reanalysis, with data visualisations produced using IDL 8.3 (Exelis Visual Information Solutions, Boulder, Colorado).

**Supplementary Figure 15 | The influence of ENSO on lightning and thunderstorm characteristics over eastern China, for the time period from 1996 to 2013.** The region is bounded by the red box shown in the map (upper panel) and examined here for DJF (a), MAM (b), JJA (c) and SON (d). Daily lightning flash density versus NINO3.4 is shown (left panels: red asterisks for 1998), with median values of lightning flash density (horizontal dotted lines) and median values of NINO3.4 (vertical dotted lines). Correlation coefficients are shown (right panels, 'x' symbols) for the relationships between NINO3.4 and three atmospheric parameters (based on reanalysis) associated with thunderstorm occurrence: specific humidity (SH), temperature lapse (TL) and convective available potential energy (CP). Correlation coefficients are also shown (right panels, '□' symbols) for the relationships between lightning flash density and the three atmospheric parameters. Coastlines are shown based on the land-sea mask of the ERA-Interim reanalysis, with data visualisations produced using IDL 8.3 (Exelis Visual Information Solutions, Boulder, Colorado).
